# Wearable microPosters

**DOI:** 10.17912/micropub.biology.000192

**Published:** 2019-11-26

**Authors:** James S Lee, Paul W Sternberg

**Affiliations:** 1 Division of Biology and Biological Engineering, Caltech; 2 Rockefeller University

**Figure 1.**
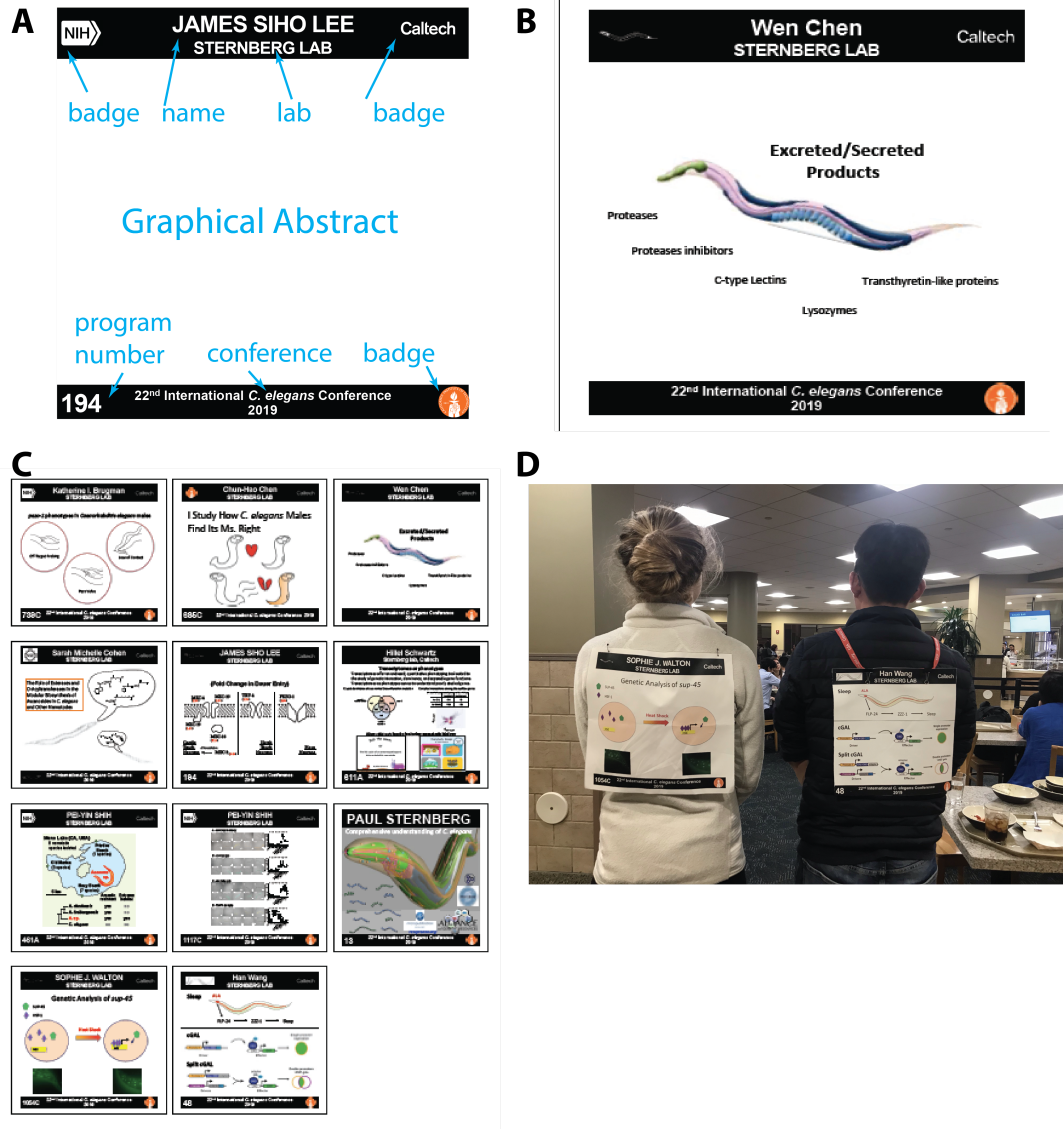
**Figure 1**. A. An example layout of the bib. B. An example of a microPoster. C. Example of a cloth poster layout prior to cutting. D. photographs of microPosters in action. 22^nd^ International C. elegans Meeting, UCLA, June 2019.

## Description

One barrier to communication at large face-to-face meetings, especially those focusing on scientific or technical topics, is a rapid way to understand what a person knows and thinks about before initiating a conversation. We have devised and reduced to practice an effective way to accomplish this task, the wearable microPoster.

In our experience, we observed that at most scientific meetings, especially once the number of participants passed 100, it is difficult to get productive conversations started with strangers. Thus, conversations tend to initiate among people that already know one another. This tends to exclude newer participants and those who do not have formal speaking slots. The difficulty increases at informal aspects of meetings: coffee breaks, lines for meals, social events, outdoor events in which a prominent speaker might have different clothes and sunglasses and is thus rendered unknown.

Poster sessions have long provided a venue for rapid communication and finding participants. These are usually the most productive parts of meetings. Our thinking evolved from jokes about sandwich board posters, in which one walks around with large posters on the front and back, to shirts with printed information. The advent of printed cloth posters provides a practical solution in which a microPoster is cut from the larger poster (ideally cloth), or printed on one or more sheets of standard paper, which are then taped together. A microPoster comprises your name, affiliation (with appropriate granularity determined by the meeting), and a graphical abstract. The goal is to identify yourself in a much more informative way than a nametag.

One convenient way to present the microPoster is pinned on your back like a bib number (e.g., as in marathons or dances). We carried out a pilot with ten microPosters at a meeting of 1500 people (Figure 1), and found them to be effective at attracting useful conversation.

The addition of a machine readable QR code (e.g., Salt, 2019) is a possible useful addition but we note that the expense in time, money and carbon footprint of a face-to-face meeting is better spent on face-to-face conversations.
